# Procedures for transferring organizational knowledge during generational change: A systematic review

**DOI:** 10.1016/j.heliyon.2024.e27092

**Published:** 2024-02-24

**Authors:** Elene Igoa-Iraola, Fernando Díez

**Affiliations:** Universidad de Deusto, Spain

**Keywords:** Knowledge transfer, Knowledge management, Generational change, Business development, Systematic review

## Abstract

The loss of organizational knowledge has emerged as a prevalent issue for 21st-century organizations. This systematic review aims to scrutinize knowledge transfer procedures applied to individuals in managerial and intermediate positions during generational change or knowledge management initiatives. Following the PRISMA statement [1], this review progressed through four stages, applying inclusion and exclusion criteria, and ultimately identifying 28 articles for the final analysis. Descriptive indicators and content-related metrics were employed in the study. Key findings include: (1) predominant investment in knowledge retention studies and procedure design is observed among large companies, primarily in the secondary sector; (2) digitalization emerges as a critical aspect of effective organizational knowledge transfer procedures and protocols; (3) intra-organizational communication styles are predominantly employed for knowledge transfer; (4) organizations prefer a collective approach to transferring both tacit and explicit knowledge. In summary, this research offers fresh insights into a pivotal area of business management, showcasing originality in its exploration of knowledge transfer within the realms of generational change and knowledge management.

## 1 Introduction

The significance of knowledge within organizations has consistently been acknowledged, but its importance has notably heightened in recent years, establishing it as a central axis in organizational dynamics [1]. The continual evolution of the labor market requires organizations to adapt their priorities and strategies to enhance organizational efficiency [2]. Thus, the research question of this study is to analyze what knowledge transfer procedures are applied to employees in managerial and intermediate positions to carry out generational changes on knowledge management. The main objectives of this research are to identify which are the companies that invest the most on designing procedures or tools for knowledge retention, to analyze the role of digitalization on organizational knowledge transfer procedures, to analyze the communication styles used for knowledge transfer procedures and to identify the type of knowledge used in organizations.

Organizations are now grappling with the repercussions of the demographic decline prevalent in recent years [3]. Currently, no country within the EU-27 achieves the fertility rate of 1.53 children per woman, a benchmark essential for ensuring generational replacement and population stability [4]. As a result, the labor market is witnessing a decline in the influx of new workers, attributed to the diminishing birth rates over the past decades [5]. Indeed, from 2023 onwards, there will be an increase in retirements of active workers belonging to the baby boom generation [6]. Projections indicate a shortfall in manpower and talent to fill the resulting vacancies, posing a significant challenge [7]. This generation's retirement is going to impact on all sector of the market, exerting a direct influence on private and public enterprises' productivity and organizational efficiency [8].

The most crucial resource of an organization is human capital, understood as the practical, theoretical, technical knowledge and skills that employees develop over years within the organization [9]. Despite the pivotal nature of workers' knowledge, a deficiency in strategies, procedures, and instruments for knowledge transfer exists in most organizations [10]. Consequently, this research scrutinizes knowledge transfer procedures applied to managerial and middle management workers involved in generational change processes or knowledge management processes. The significance of this study lies in the applicability of the results for further research studies in the field and for practitioners. Enterprises can benefit from the contributions made in this study as human resources practices or knowledge management strategies could be implemented in organizations. The application of scientific results to the enterprises improves the efficiency and competitiveness of organizations [11]. The uniqueness of this study lies in its innovative contribution to the exploration of a relatively under-researched topic, specifically the analysis of generational change processes and the procedures employed for knowledge management in the organizational realm. The outcomes of this research highlight the attributes and characteristics that are required for a procedure to successfully transfer knowledge from one employee to another, thereby facilitating the retention of knowledge within the organization.

Knowledge management provides insights into creating, acquiring, sharing, and utilizing knowledge within organizations [12]. Several scholars underscore knowledge management as pivotal in ensuring organizational success and competitiveness [13,14]. Effectively managing knowledge requires consideration of various organizational factors, including HR resources, intellectual capital, management practices, organizational strategy and structure, and technological resources [15]. A comprehensive approach to knowledge management requires an analysis of the organization's internal and external context.

Digitalization is essential in knowledge management [16], wherein digital technologies reshape social or organizational contexts by implementing digital communities and virtual infrastructures [17,18]. These transformations impact the organizationś and employeeś resources or procedures to employ daily tasks as well as it entails changes in social aspects, including communication methods [19]. In recent years, there has been an exponential increase in the adoption of digital innovation strategies by both public and private entities [20,18]. This can be interpreted as an organizational response to the imperative of sustaining competitiveness in the labor market [21]. Substantial disparities in competitiveness are evident between organizations that have embraced digitalization and those that have not [22]. Adapting to the digital environment is also prompted by the generational diversity prevalent in most organizations [23]. Within organizations, up to four generations (Baby Boomers, Generation X, Millennials, and Generation Y) may coexist, each exhibiting distinct orientations toward work, the organization, and communication styles [24]. In recent years, various approaches have been introduced to facilitate bidirectional communication and learning among different generations [25,26]. The coexistence of generations should be considered in the realm of knowledge management but also in the knowledge transfer procedures. Regardless of their generation, workers possess valuable knowledge, crucial for organizational success that must be retained [27].

Knowledge transfer can be executed through various procedures, contingent on the internal and external context of the organization [28]. Studies suggest that the level of trust and the relationships among workers significantly influence knowledge exchange [29]. Moreover, the crucial role of managers and middle managers in knowledge transfer cannot be overstated, as they serve as key figures in creating a collaborative context within the organization and among workers involved in knowledge transfer [30]. Knowledge transfer may occur within or between departments, with other organizations [31], or in cross-border contexts [32]. The extent and nature of transfer depend on organizational management, strategies, and characteristics [33,34]. Indeed, research indicates that organizations engaging in more activities related to employee coordination, soft and technical skills, or those that foster continuous learning tend to be more successful in knowledge transfer [35,36].

Regarding the type of knowledge that can be transferred, a distinction can be made between tacit and explicit knowledge [37]. Tacit knowledge is characterized by workers’ cognitive abilities, including ideas, mental models, know-how, personal skills, or intuition, developed over time through specific practices [38]. It is deeply embedded in the individual and reflects their personal way of working [39]. On the other hand, explicit knowledge typically belongs to the organization and can be codified. The transfer of explicit knowledge generally occurs formally through documents, reports, procedures, manuals, or computer applications, making the transfer process more straightforward [40]. Recognizing these two types of knowledge is crucial for effective knowledge management and transfer, as it facilitates the development of a well-designed approach to knowledge retention [41,42]. The process of generational change typically unfolds among experienced employees with expertise who are transitioning into retirement [3].

This transition involves transferring theoretical and practical knowledge, including essential skills and know-how, to incoming employees intending to continue their organizational tenure. The objective of such transitions is to establish replacement strategies and facilitate knowledge transfer, ensuring that the departure of experienced individuals does not compromise the quality of organizational production [43]. There are different strategies to carry out generational change processes within organizations, but they all share a common goal: preserving departing employees' knowledge and essential skills. These strategies aim to enable the seamless continuation of tasks without compromising organizational competitiveness [44]. Through generational change, organizations can effectively retain organizational knowledge by ensuring the transfer of necessary competencies, thereby averting organizational loss and sustaining business competitiveness [45]. The transfer of skills and knowledge can be understood as a productive factor within the organization, as it gives value to employees and organizations [15].

Recognizing that the transmission of skills and knowledge is elemental for organizational benefit, companies are adopting various strategies to extract and analyze the diverse ways of knowledge transfer among employees [44,46]. Identifying key positions is essential to this process, as not all employees possess the same knowledge, and therefore, their absence may vary in the impact of the organization [47]. Simultaneously, consideration must be given to each job's diversity and distinct characteristics, which can vary across sectors, markets, or companies [48]. Jobs may differ in specific cognitive, physical, technical, or social aspects, demanding precision in procedural design [49]. Consequently, challenges arise from the difficulty of pinpointing specific knowledge and key competencies of a position, and the formulation of specific procedures.

Hence, we deem it imperative to undertake a thorough investigation focusing on analyzing procedures for knowledge transmission within the realm of knowledge management. The research's objective is to conduct a systematic review elucidating the current state of procedures for knowledge transfer in knowledge management, particularly within generational change processes. Consequently, this study signifies a significant advancement in understanding this domain, given the escalating interest in its diverse applications in recent years, spanning public and private organizations across various sectors. The results obtained in this research are a step forward for companies that want to improve their knowledge transfer strategies as different practical strategies are presented. Moreover, this study gives opportunity for further research investigations on the field of knowledge management and generational change. The study's outcomes delineate the most commonly employed procedures in knowledge management, clarifying the essential factors for achieving effective knowledge transfer within organizations.

## 2 Methodology

The PRISMA (Preferred Reporting Items for Systematic Reviews and Meta-Analyses) statement was employed to enhance this study's transparency, coherence, and comprehensiveness. Widely adopted across numerous journals, this methodology boasts over 60,000 citations in reports and is endorsed by more than 200 journals and organizations engaged in systematic reviews across diverse disciplines [50]. Simultaneously, the PICO framework was utilized for an objective and impartial determination of study selection criteria [51]. To amalgamate and interpret the findings from the articles included in this systematic review, the work of Lockwood et al. [52], served as a foundation. Furthermore, adherence to the 11 generic criteria outlined by the Joanna Briggs Institute (JBI) [53] assures the transparency, validity, and replicability of the conducted systematic review.

### 2.1 Research objectives and question

The research question of this systematic review has been carried out following the guidelines of the PICO strategy determined by the National Institute for Health and Care Excellence [51], endorsed in the Cochrane Handbook for Systematic Reviews of Interventions [54]. The PICO acronym consists of the following terms: population (P); intervention (I); comparison, control, or comparator (C); and result (O). Thus, the research question and main objective are to answer the following question: What knowledge transfer procedures (I) are applied with employees in managerial and intermediate positions (P) to carry out generational change or knowledge management (O)? In this case, comparison (C) was not applied since different populations are not compared.

### 2.2 Search strategy

This research adheres to the established steps of the PRISMA declaration, ensuring a rigorous, transparent, and reproducible systematic review to mitigate bias in data collection and furnish scientific evidence on the designated research topic. The search was conducted during the months of June and July 2023 on databases such as Web of Science (WoS), Scopus, ProQuest One Business, and Business Source Complete (Ebsco). For inclusion in this review, only articles published in journals, subjected to expert evaluation, and written in English were considered, excluding book chapters, reports, or proceedings of scientific conferences. The search for articles was conducted using keywords established following the PICO strategy (Table 1).

**Table 1:** Keywords formulated with the PICO strategy.

	[50] Population	[1] Intervention	[2] Results
Keywords	“Managerial” OR “Manager” OR “CEO” OR “Executive” OR “Director” OR “Middle management” OR “Intermediate staff”	“Knowledge transfer” OR “Knowledge transfer practice” OR “Knowledge sharing” OR “Knowledge sharing procedure”	"Succession management" OR "Knowledge management" OR "Succession planning" OR Generational change” OR “Generational handover” OR “Generational replacement” OR “Generational relay” OR “Generational succession”
Searches	**WoS**: TOPIC [50] AND TOPIC [1] AND TOPIC [2] **Scopus:** TITLE-ABS-KEY [50] AND TITLE-ABS-KEY [1] AND TITLE-ABS-KEY [2] **ProQuest One Business:** NOFT = [50] AND [1] AND [2] **Business Source Complete (Ebsco):** (TI [50] OR SU [50] OR AB [50]) AND (TI [1] OR SU [1] OR AB [1]) AND (TI [2] OR SU [2] OR AB [2])		

### 2.3 Inclusion and exclusion criteria

To conduct an unbiased research endeavor, the establishment of inclusion and exclusion criteria is imperative. The PICO strategy has been employed in this instance, as depicted in Table 2 [55]. This review includes articles with qualitative, quantitative, and mixed designs and excludes literature review studies.

**Table 2:** Inclusion and exclusion criteria formulated with the PICO strategy.

	Population	Intervention	Results
**Inclusion criteria**	Participants who belong to the business world and who Have key positions within the organization (managerial or intermediate positions).	Strategies for knowledge retention through knowledge transfer, management, or exchange.	They report on the success of the generational change or PICO knowledge management
**Exclusion criteria**	Participants who do not belong to an organization. Participants who do not belong to key positions within the organization.	Studies where no type of strategy or procedure is carried out.	Do they report on other unrelated generational changes or knowledge management details?

### 2.4 Selection process

**Fig. 1:**
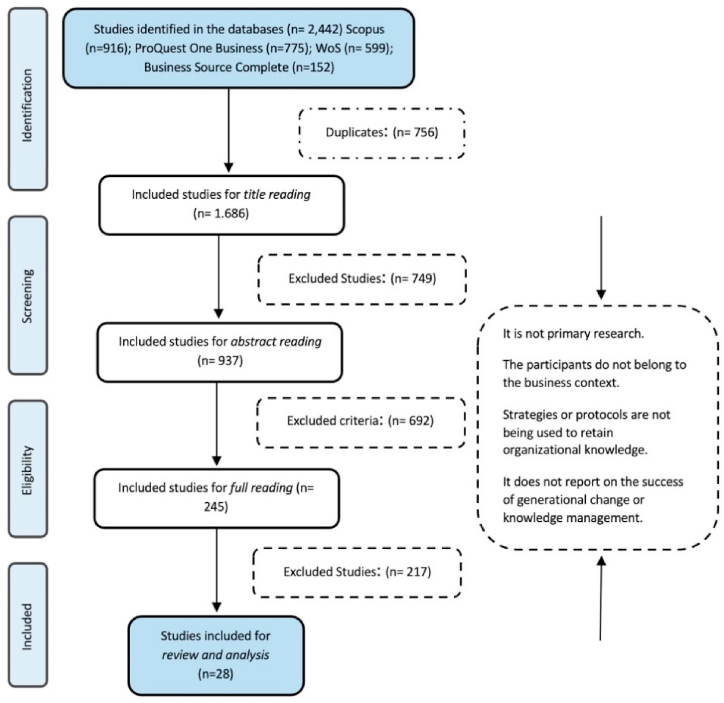
Study selection procedure flowchart.

Source: Own elaboration

The study selection process involves several phases, with collaborative efforts from the researchers who authored this article (Fig. 1). In the initial phase, 2442 studies were identified in the Scopus, ProQuest One Business, Web of Science (WoS), and Business Source Complete databases. Bibliographic references were exported to Rayyan, a collaborative web application for systematic reviews [56]. Through this platform, 756 duplicate documents were identified and subsequently eliminated. The outcome revealed a total of 1686 studies for comprehensive review.

In the screening phase, adhering to the inclusion and exclusion criteria, the titles of the initial 1686 articles were scrutinized. Consequently, 749 investigations that did not align with the inclusion criteria were excluded. Following this, a similar process was executed with the abstracts of the remaining 937 studies, resulting in the exclusion of 692 studies. The culmination of this stage yielded a total of 245 articles earmarked for further analysis.

Moving into the selection phase, the full text of the 245 studies was independently reviewed, excluding 217 studies that did not meet the established inclusion and exclusion criteria. Consequently, the final result after this rigorous process is a total of 28 articles, which underwent subsequent detailed review and reading.

### 2.5 Quality assessment

To affirm the validity and impact of the final studies selected for the systematic review, the CASP (Critical Appraisal Skills Programme) evaluation criteria checklist was employed [57]. This tool was chosen because it is widely used for evaluating the quality of qualitative evidence syntheses and is supported by the Cochrane Qualitative and Implementation Methods Group [58]. The evaluation consists of three phases in which the validation of the selected articles, the quality of the results obtained, and the impact of the results are analyzed. Even though CASP is one of the most used programs for stabilizing evaluation criteria in systematic reviews, some of its limitations could be related to researchers' biases.

### 2.6 Data analysis

To gain a comprehensive understanding of the subject under investigation, it is deemed essential to analyze organizations’ characteristics, including the procedures and types of knowledge addressed in the selected studies. To systematize the acquired information, several variables have been selected, encompassing geographical factors (country where the study was conducted), organizational size (large, medium, small), sector (primary, secondary, tertiary), methodological aspects (research objectives, results, methods, characteristics, instruments), procedural details (procedure characteristics, level of digitalization), organizational communication (intra-organizational, interorganizational), and knowledge-related factors (knowledge characteristics). Two complementary tools have been employed for the systematic organization and analysis of data: Excel and Rayyan spreadsheets [56]. Rayyan, a collaborative and validated tool [59], was particularly beneficial in facilitating collaboration among authors throughout the article selection process for the review. It is a frequently utilized tool in systematic reviews.

## 3 Results

To analyze the 28 articles obtained through the systematic review employing the PRISMA methodology, descriptive data from the studies, and information on their content will be presented.

### 3.1 Descriptive analysis of the literature under study

The analyzed studies were found in 17 countries. The country with the highest number of studies on the research topic was the United States (6 of 28), followed by France (4 of 28), Canada (4 of 28), and Taiwan (3 of 28). Some studies obtained samples in different countries such as Nigeria and the United Kingdom, Europe and the USA, or Portugal, Spain, France, and Italy (Table 3, Table 4).

**Table 3:** Descriptive data of the analyzed organizations.

Autor(s)	Location	Company size: Big (B), Medium (M), Small (S)	Company Industry
Leon [60]	RO	–	Hotel
Sungkur & Ramasawmy [61]	–	–	Software industry
Jørgensen et al. [62],	DE	–	Healthcare
Maravilhas & Martins [63]	PO; ES; FR; IT	–	STEM
Jackson [64]	AU	B	–
García et al. [65],	FR	–	Manufacturing
Mariano & Awazu [66]	US	M	Technology
Vaz-Serra & Edwards [67]	PO	–	Engineering and Construction
Patalas-Maliszewska [68]	PL	–	Manufacturing
Mahura, A., & Birollo [69]	CA	B	Provincial Government Agency
Arfi et al. [70],	TN	S	Dairy industry
Afshar Jalili [71]	IR	–	Oil Industry
Curtis & Taylor [72]	US	–	Public Accounting
Perrigot et al. [73]	FR	–	Clothing, Beauty, Specialized food, Fast food, Retailing
Gamo-Sanchez & Cegarra-Navarro [74]	ES	S	Engineering & Maintenance
Chigada & Ngulube [75]	ZA	–	Banking
Ho & Kuo [76]	TW	–	Human Resources
Zhao & Chen [77]	CN	B	Computer hardware & Software
Sanaei et al. [78],	US	B	Construction & Engineering
Ho et al. [79],	TW	–	Construction
Wang, & Wei [80]	TW	–	–
Fannoun & Kerins [81]	UK	–	–
Ochieng et al. [82],	UK; NG	–	Oil & Gas
Neeley et al. [83],	US	B	Technology and Banking
Nazemi et al. [84],	IR	–	Automation
Hutzschenreuter & Horstkotte [85]	US; EU	B	–
Roth [86]	SE	B	Pharmaceutical
Landry et al. [87],	CA	B	Education

**Table 4:**
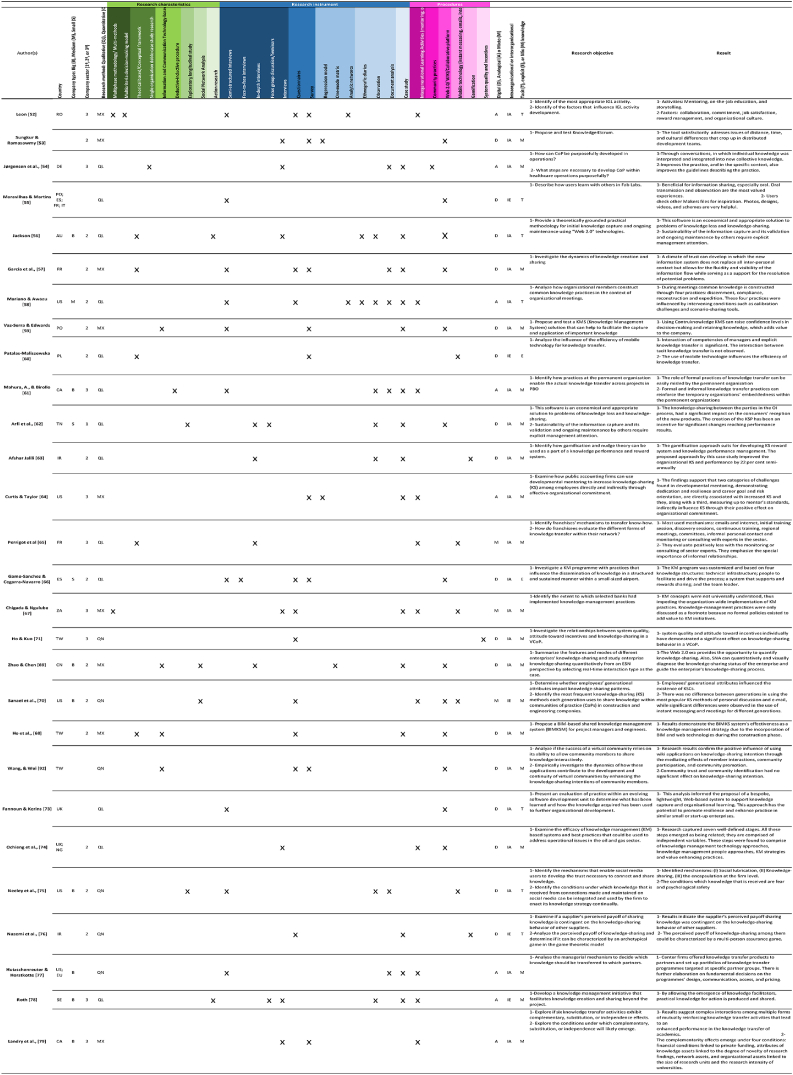
Variable matrix.

Considering that the primary objective of this review is to analyze procedures for knowledge retention, it is intriguing to explore the sizes of the companies involved in the analyzed studies. This consideration arises from studies indicating a relationship between organizational size and investment in Research and Development (R&D). Notably, most organizations in the studies do not specify their size within the articles (17 out of 28). Among those that do specify, 82% are identified as large companies, while 18% fall into the category of medium-sized companies. Interestingly, no organization in the study is classified as a small business (Table 3, Table 4).

A categorization has been conducted to identify the most frequently used types of interviews. Semi-structured interviews are the most prevalent (10 out of 28), followed by open interviews (7 out of 28). In third place are in-depth interviews (4 out of 28), followed by group interviews, also referred to as focus groups or seminars (2 out of 28). Lastly, face-to-face interviews are the least commonly used (1 out of 28). Consequently, the majority of interviews have been conducted individually, although group interviews have also been identified. Within the realm of qualitative instruments, observation techniques have been utilized in 7 out of 28 studies, followed by the analysis of documents used in the organization for knowledge management (6 out of 28) and ethnographic diaries (2 out of 28).

The second most frequently used research instrument is questionnaires (10 out of 28), followed by surveys (8 out of 28). In most studies, these qualitative instruments have been combined with quantitative techniques. The identified quantitative techniques in this review include regression models (2 out of 28), analytical reviews (2 out of 28), and unimodal matrixes (1 out of 28). Finally, it is noteworthy that 53% of the analyzed studies were case studies.

### 3.2 Content analysis for the studies

In the upcoming section, we will delve into the content of the selected studies, considering the following axes: the type of procedure, the level of procedural digitalization, organizational communication styles in knowledge transfer, and the type of knowledge transmitted through these procedures. This analysis aims to provide a comprehensive understanding of the characteristics of procedures used in the organizational context for knowledge transfer, with the ultimate goal of promoting knowledge management.

Regarding the type of procedure, the most frequently used are Web 2.0 or WIKIS, followed by intergenerational learning activities, encompassing mentoring sessions, on-the-job training, storytelling, department meetings, creation of teams, succession plans, lunch break talks, seminars, and extensions of retirements. In third place is mobile technology, understood as instant messaging, emails, or internal social networks. Gamification is in fourth place, and incentive systems and community practices are in last (See Table 4).

Considering the type of procedure organizations employ for knowledge transfer in this study, it is evident that most companies (61%) utilize digital procedures, while 28% employ analog procedures or techniques. The procedures of the remaining 11% are characterized by using mixed tools (digital and analog). Shifting the focus to organizational communication style, it is observed that 79% of organizations tend to transfer and share organizational knowledge internally, either between departments or among staff. It can be noted that only 21% of companies share their knowledge or information with other organizations, whether they are in the same sector or not (See Table 4).

We consider it is necessary to mention that 68% of organizations transfer tacit and explicit knowledge using procedures for knowledge management. 25% of organizations transfer tacit knowledge, and only 7% consider it necessary to transfer just explicit knowledge (See Table 4).

## 4 Discussion

This systematic review analyses the procedures used to transfer organizational knowledge in the processes of generational change or knowledge management. The following conclusions are derived based on the 28 studies selected in the systematic review following the PRISMA guidelines.

The selected studies converge on a common issue: the loss of organizational knowledge. This challenge is raised not only due to the aging of the organizations' workforce but also due to the absence of effective management and internal procedures within the organizations [88]. The analyzed studies address this issue by presenting diverse approaches to retain the internal knowledge of organizations, suggesting and testing different procedures. Consequently, various tools are identified and tailored to the characteristics of each organization, sector, and market. The obtained research results are generalizable to different organizational contexts and geographies as the analyzed sample was diverse. This diversity reflects the heterogeneity of the analyzed procedures.

The results show, firstly, that the companies that invest the most in conducting studies, designing procedures, or tools for knowledge retention are large-sized companies. Usually, large organizations have departments that focus on the analysis of internal resources to improve organizational performance. Indeed, the research carried out by Al Singh et al. [89], highlights the importance of practices to create and retain knowledge in middle management positions. They also have greater economic resources to invest in this type of strategy or new lines of research [90]. On the contrary, medium or small companies do not usually have these resources, even if they may be aware of the problems related to knowledge loss. In most cases, they do not have the time or financial resources to be able to address these problems [91]. When we talk about companies with the characteristics of an SME, they generally do not have HR or R&D departments that contemplate this type of problem or possible solutions [9]. In turn, in coincidence with the study carried out by Da Silva et al. [92], the results in our research confirm how those companies belonging to the secondary sector tend to invest more in this type of protocols and procedures to solve the problem of knowledge transfer and knowledge retention within organizations. Thus, we can observe that different external characteristics, such as the organization's size and sector, can make a difference in business competitiveness within the labor market.

Secondly, the analyzed studies highlight that a crucial characteristic of organizational knowledge transfer procedures is the digitalization. This trend can be attributed to the global digital transformation that all organizations are currently undergoing [93]. Indeed, the research carried out by Ribeiro-Navarrete [94] shows that having digital resources and high level of training in digital tools help managers enhance company performance. Digital procedures streamline the recording of searches, usage instructions for tools, or process monitoring, as everything is documented [95]. This may facilitate the implementation of procedures. Additionally, adapting internal procedures within organizations may be influenced by the diversity of generations in the workforce [96]. In cases of generational change, it is advisable for knowledge transfer procedures to be tailored to the younger generations in the workforce, as they will need the transferred knowledge [97]. Despite the existence of analog procedures for knowledge transfer, the research indicates that 89% of the analyzed studies utilize digital or mixed (digital and analog) procedures or protocols. Hence, it can be inferred that the prevailing trend among organizations is to adopt fully digital or partially digital procedures. Once again, this underscores the distinction between organizations proactively embracing digitalization and those that do not. Organizations lacking a digital infrastructure will likely face challenges in creating digital procedures for transferring organizational knowledge.

Thirdly, the studies underscore that the predominant communication style used for knowledge transfer is intra-organizational. On the study carried out by Zenk et al. [98], the results highlighted the intra-organizational knowledge sharing behavior where identified when analyzing employees’ attitudes, organizational support and specific relational aspects. This preference is attributed to the faster creation, approval, and implementation of a protocol or procedure within a single organization, as there is alignment with organizational objectives, mission, and values [99]. Some studies suggest that transferring information between departments within an organization is easier than doing so between different organizations, mainly due to issues of trust and competitiveness [100]. In the reviewed literature, studies such as that by Gervais et al., [101]. were identified, which discuss procedures for transferring knowledge between departments in different countries.

Fourthly, it is observed that most organizations prefer to transfer both tacit and explicit knowledge collectively. This preference stems from recognizing that both types of knowledge are valuable, necessary, and contribute to organizational competitiveness across all roles. However, tacit knowledge poses a greater challenge in terms of transmission, as it involves the worker's know-how, which can be more challenging to articulate and internalize [102]. It is noteworthy that in several studies, analyzed by Cairó and Bork [103] and Nupap [104], the emphasis was on the objective of procedures being tested or used to convert tacit knowledge into explicit knowledge. This approach aims to facilitate knowledge transfer in generational change processes within the organization, thereby sustaining business competitiveness.

This research has successfully achieved its objective of analyzing knowledge transfer procedures in knowledge management for generational change processes. However, certain limitations have been identified, such as excluding articles not written in Spanish or English. Future reviews may benefit from considering texts in other languages. Another limitation is that the analyzed studies belong to journals indexed in the WoS, Scopus, ProQuest One Business, and Business Source Complete (Ebsco) databases. Future research might enhance its scope by incorporating additional databases to gain a more comprehensive perspective on the subject. Future lines of research should explore smaller enterprises or different sectors in order to obtain a general overview of the market in terms of knowledge retention strategies. Indeed, it would be enriching comparing different geographically located enterprises knowledge sharing practices to the influence of different contextual factors. Lastly, it is important to note the challenges encountered in identifying studies specifically addressing generational change. This suggests that the concept of generational change is relatively new in the field of business research.

## 5 Conclusions

In conclusion, the conducted systematic review provides a nuanced understanding of the scientific production concerning research on procedures for knowledge transfer in organizational management within generational change processes. Firstly, the research highlights that large companies invest more in conducting studies and formulating procedures for knowledge retention during generational change processes. This underscores the vulnerability of medium and small companies to the loss of organizational knowledge, potentially impacting their competitiveness in the labor market. Secondly, the study emphasizes the pivotal role of digitalization in achieving excellence in knowledge transfer procedures. Thirdly, the review underscores the significance of communication in knowledge management, revealing that most organizations rely on intra-organizational communication channels. Finally, the research delineates the types of knowledge (tacit and explicit) deemed essential for transfer, ensuring the preservation of relevant knowledge during generational change processes.

Considering the data analyzed on this research, organizations should invest on new channels of digitalization to improve knowledge transfer practices that secure tacit and explicit knowledge. This would give new strategies to retain knowledge and strengthen new procedures to employeeś internal communication guaranteeing unity of the workforce. In the research field, more attention should be paid to the heterogeneity of the companies as significant differences can be identified depending the organization characteristics such as the size and the resources to invest on knowledge sharing procedures.

The originality of this study lies in its unique focus, as there are limited reviews conducted to date on the specific topic of procedures for knowledge transfer within generational change processes. Additionally, this research represents a significant advancement for the development of generational change procedures, providing valuable insights for structuring effective processes. The study offers essential characteristics to guide the creation and analysis of knowledge transfer procedures for robust organizational knowledge management. This research fills a gap in the existing literature and serves as a foundational study providing insights for both the scientific and business communities. The findings further the understanding of global knowledge management realities in organizations, offering valuable input to refine and strengthen established organizational strategies. Furthermore, the study's outcomes can potentially enhance organizational strategies and procedures for knowledge transfer, thereby improving overall organizational performance, competitiveness, and efficiency.

## Funding information

This research is funded by the 10.13039/501100019927University of Deusto (Univesidad de Deusto). FPI Call (Formación de Personal Investigador).

## Data availability statement

No data associated to this study has been deposited into a publicly available repository.

Data will be made available on request.

## Ethics declaration

This study was reviewed and approved by *The Research Ethics Committee of the University of Deusto*, with the approval number: ETK-22/23-24.

## CRediT authorship contribution statement

**Elene Igoa-Iraola:** Writing – review & editing, Writing – original draft, Methodology, Investigation, Conceptualization. **Fernando Díez:** Supervision, Formal analysis.

## Declaration of competing interest

The authors declare that they have no known competing financial interests or personal relationships that could have appeared to influence the work reported in this paper.

## References

[1] Manesh M.-F., Pellegrini M.-M., Marzi G., Dabic M. (2020). Knowledge management in the fourth industrial revolution: mapping the literature and scoping future avenues. IEEE Trans. Eng. Manag..

[2] Errida A., Lotfi B. (2021). The determinants of organizational change management success: literature review and case study. Int. J. Eng. Bus. Manag..

[3] Helal D.-H., Nóbrega C.-V., Lima T. (2021). Retirement and organizations: perspectives and challenges for both workers and human resource management. Work. Older People.

[4] Eurostat Fertility Statistics (2021). https://ec.europa.eu/eurostat/statistics-explained/index.php?title=Fertility_statistics.

[5] European Commission (2014). https://data.europa.eu/doi/10.2777/60452.

[6] European Commission (2020).

[7] Brunello G., Wruuck P. (2021). Skill shortages and skill mismatch in Europe: a review of the literature. J. Econ. Surv..

[8] Bixby R.L. (2020). Impacts of aging on the federal budget and economy: a cross-cutting challenge. Public Policy & Aging Report.

[9] Jooss S., Burbach R., Ruël H. (2021). Examining talent pools as a core talent management practice in multinational corporations. Int. J. Hum. Resour. Manag..

[10] Cox V., Overbey J.-A. (2023). Generational knowledge transfer and retention strategies. Dev. and Learning in Organizations.

[11] Ode E., Ayavoo R. (2020). The mediating role of knowledge application in the relationship between knowledge management practices and firm innovation. J. Innov. & Know..

[12] Obeidat B.-Y., Al-Suradi M.-M., Masa’deh R.-E., Tarhini A. (2016). The impact of knowledge management on innovation: an empirical study on Jordanian consultancy firms. Manag. Res. Rev..

[13] Xue C.-T.-S. (2017). A literature review on knowledge management in organizations. Res. Bus. Manag..

[14] Ogutu H., El Archi Y., Dénes Dávid L. (2023). Current trends in sustainable organization management: a bibliometric analysis. Oeconomia Copernicana.

[15] Mehrez A.-A.-A., Alshurideh M., Kurdi B.-A., Salloum S.-A. (2021). Proceedings of the International Conference on Advanced Intelligent Systems and Informatics 2020.

[16] De Bem Machado A., Secinaro S., Calandra D., Lanza Lužar a F. (2022). Knowledge management and digital transformation for Industry 4.0: a structured literature review. Knowl. Manag. Res. Pract..

[17] Baskerville R.-L., Myers M.-D., Yoo Y. (2019). Digital first: the ontological reversal and new challenges for IS research. MIS Q..

[18] Barker R. (2015). Management of knowledge creation and sharing to create virtual knowledge-sharing communities: a tracking study. J. Knowl. Manag..

[19] Cascio W.-F., Montealegre R. (2016). How technology is changing work and organizations. Annual Rev. of Organizational Psychology and Organizational Behav..

[20] Alvarenga A., Matos F., Godina R., Matias J. (2020). Digital transformation and knowledge management in the public sector. Sustainability.

[21] Wiesböck F., Hess T. (2020). Digital innovations: embedding in organizations. Electron. Mark..

[22] Menz M., Kunisch S., Birkinshaw J., Collis D.-J., Foss N.-J., Hoskisson R.-E., Prescott J.-E. (2021). Corporate strategy and the theory of the firm in the digital age. J. Manag. Stud..

[23] Meret C., Fioravanti S., Iannotta M., Gatti M. (2018).

[24] Lyons S., Kuron L. (2014). Generational differences in the workplace: a review of the evidence and directions for future research. J. Organ. Behav..

[25] Richards M.-B., Becker K.-L., Stollings-Holder J. (2023). Escaping generational conflict: using gamification to examine intergenerational communication & problem-solving. Rev. of Managerial Sci..

[26] Chih W.-H., Huang L.-C., Yang T.-J. (2016). Prior knowledge, transformative learning and performance. Ind. Manag. Data Syst..

[27] Ahmad F., Karim M. (2019). Impacts of knowledge sharing: a review and directions for future research Journal of Workplace Learning.

[28] Gaur A.-S., Ma H., Ge B. (2019). MNC strategy, knowledge transfer context, and knowledge flow in MNEs. J. Knowl. Manag..

[29] Curado C., Vieira S. (2019). Trust, knowledge sharing and organizational commitment in SMEs. Person. Rev..

[30] Vlajčić D., Caputo A., Marzi G., Dabić M. (2019). Expatriates managers' cultural intelligence as promoter of knowledge transfer in multinational companies. J. Bus. Res..

[31] Xu J., Wang C., Cui Y. (2023). Multidimensional proximities and interorganizational coinnovation performance: the roles of intraorganizational collaboration network inefficiency. Front. Psychol..

[32] Sheng M.-L., Hartmann N.-N. (2019). Impact of subsidiaries' cross-border knowledge tacitness shared and social capital on MNCs' explorative and exploitative innovation capability. J. Int. Manag..

[33] Bacon E., Williams M.-D., Davies G.-H. (2019). Recipes for success: conditions for knowledge transfer across open innovation ecosystems. Int. J. Inf. Manag..

[34] Brix J. (2017). Exploring knowledge creation processes as a source of organizational learning: a longitudinal case study of a public innovation project. Scand. J. Manag..

[35] Al Nahyan M.-T., Sohal A., Hawas Y., Fildes B. (2019). Communication, coordination, decision-making and knowledge-sharing: a case study in construction management. J. Knowl. Manag..

[36] Cohen J.-F., Olsen K. (2015). Knowledge management capabilities and firm performance: a test of universalistic, contingency and complementarity perspectives. Expert Syst. Appl..

[37] Collins H.M. (2010).

[38] Collins H.M. (2005). The Practice Turn in Contemporary Theory.

[39] Nonaka I., Takeuchi H. (1997).

[40] Wang Z., Sharma P.N., Cao J. (2016). From knowledge sharing to firm performance: a predictive model comparison. J. Bus. Res..

[41] Chilton M.-A., Bloodgood J.-M., Management Association I. (2012). Organizational Learning and Knowledge: Concepts, Methodologies, Tools and Applications.

[42] Abubakar A.-M., Elrehail H., Alatailat M.-A., Elçi A. (2019). Knowledge management, decision-making style and organizational performance. Journal of Innov. & Knowl..

[43] Clarke A.J., Burgess A., van Diggele C., Mellis C. (2019). The role of reverse mentoring in medical education: current insights. Adv. Med. Educ. Pract..

[44] Tavasieva Z., Pozmogov A., Kreer M., Kallagov B., Tedeyeva Z. (2019). Knowledge management as a matter of vital importance for a modern organization, education excellence and innov. Manag. Through Vision.

[45] Pandiyan A.-V.-R., Jayalashmi P. (2016). Succession management at a manufacturing company in Chennai: an empirical study. TSM Bus. Rev..

[46] Ali A., Bahadur W., Wang N., Luqman A., Khan A.-N. (2020). Improving team innovation performance: role of social media and team knowledge management capabilities. Technol. Soc..

[47] Li H., Hausknecht J.-P., Dragoni L. (2020). Initial and longer-term change in unit-level turnover following leader succession: contingent effects of outgoing and incoming leader characteristics. Organ. Sci..

[48] Hauret L., Williams D.-R. (2020). Workplace diversity and job satisfaction. Equal. Divers. Incl..

[49] Seo H. (2021). Dual’labour market? Patterns of segmentation in European labour markets and the varieties of precariousness” Transfer. Eur. Rev. of Labour and Res..

[50] Page M., Moher D., Bossuyt P.-M., Boutron I., Hoffmann T., Mulrow C.-D. (2021). PRISMA 2020 explanation and elaboration: updated guidance and exemplars for reporting systematic reviews. Br. Med. J..

[51] Schardt C., Adams M.-B., Owens T., Keitz S., Fontelo P. (2007). Utilization of the PICO framework to improve searching PubMed for clinical questions. BMC Med. Inf. Decis. Making.

[52] Lockwood C., Munn Z., Porritt K. (2015). Qualitative research synthesis: methodological guidance for systematic reviewers utilizing meta-aggregation. Int. J. Evid. Base. Healthc..

[53] Johana Briggs Institute (2017). http://joannabriggs.org/research/critical-appraisal-tools.htm.

[54] Thomas J., Kneale D., McKenzie J.-E., Brennan S.-E., Bhaumik S., Higgins J.P.T., Thomas J., Chandler J., Cumpston M., Li T., Page M., Welch V. (2019). Determining the Scope of the Review and the Questions it Will Address.

[55] Methley A., Campbell S., Chew-Graham C., McNally R., Cheraghi-Sohi S. (2014). PICO, PICOS and SPIDER: a comparison study of specificity and sensitivity in three search tools for qualitative systematic reviews. BMC Health Serv. Res..

[56] Ouzzani M., Hammady H., Fedorowicz Z., Elmagarmid A. (2016). Rayyan — a web and mobile app for systematic Rev. Syst. Rev..

[57] Critical Appraisal Skills Programme (2022). Rev. Checkl..

[58] Long H.-A., Frenchand D.-F., Brooks J.-M. (2020). Optimising the value of the critical appraisal skills programme (CASP) tool for quality appraisal in qualitative evidence synthesis. Res. Methods Med. Health Sci..

[59] Yu F., Liu C., Sharmin S. (2022). Performance, usability, and user experience of rayyan for systematic reviews. Proc. of the Association for Info. Sci. and Technol..

[60] Leon R.-D. (2020). Fostering intergenerational learning in the hotel industry: a multiple criteria decision-making model. Int. J. Hospit. Manag..

[61] Sungkur R., Ramasawmy M. (2014). Knowledge4Scrum, a novel knowledge management tool for agile distributed teams. VINE-Journal of Inf. and Know. Manag. Systems.

[62] Jørgensen R., Scarso E., Edwards K., Ipsen C. (2019). Communities of practice in healthcare: a framework for managing knowledge sharing in operations. Knowl. Process Manag..

[63] Maravilhas S., Martins J. (2019). Strategic knowledge management in a digital environment: tacit and explicit knowledge in Fab Labs. J. Bus. Res..

[64] Jackson P. (2010). Capturing, structuring and maintaining knowledge: a social software approach. Ind. Manag. Data Syst..

[65] García F., Grabot B., Paché G. (2023). Creating and sharing interorganizational knowledge through a supply chain 4.0 project: a case study. J. Global Inf. Manag..

[66] Mariano S., Awazu Y. (2021). I hear you: constructing common knowledge practices in the context of organizational meetings. J. Knowl. Manag..

[67] Vaz-Serra P., Edwards P. (2021). Addressing the knowledge management “nightmare” for construction companies. Construct. Innovat..

[68] Patalas-Maliszewska J. (2015). The effect of the use of mobile technologies by management in polish manufacturing enterprises on the efficiency of knowledge transfer within a company. Found. of Manag..

[69] Mahura A., Birollo G. (2021). Organizational practices that enable and disable knowledge transfer: the case of a public sector project-based organization. Int. J. Proj. Manag..

[70] Arfi W., Enström R., Sahut J.-M., Hikkerova L. (2019). The significance of knowledge sharing platforms for open innovation success: a tale of two companies in the dairy industry. J. Organ. Change Manag..

[71] Afshar-Jalili Y. (2020). I rather share my knowledge: applying gamification approach and nudge theory to develop an incentive system. VINE Journal of Inf. and Know. Manag. Systems.

[72] Curtis M., Taylor E.-Z. (2018). Developmental mentoring, affective organizational commitment, and knowledge sharing in public accounting firms. J. Knowl. Manag..

[73] Perrigot R., Herrbach O., Cliquet G., Basset G. (2017). Know-how transfer mechanisms in franchise networks: a study of franchisee perceptions. Knowl. Manag. Res. Pract..

[74] Gamo-Sanchez A.-L., Cegarra-Navarro J.-G. (2015). Factors that influence the success of a KM-program in a small-sized airport. J. Knowl. Manag..

[75] Chigada J., Ngulube P. (2015). Knowledge-management practices at selected banks in South Africa. S. Afr. J. Inf. Manag..

[76] Ho L.-A., Kuo T.-H. (2013). How system quality and incentive affect knowledge sharing. Ind. Manag. Data Syst..

[77] Zhao R.-Y., Chen B.-K. (2013). Study on enterprise knowledge sharing in ESN perspective: a Chinese case study. J. Knowl. Manag..

[78] Sanaei M., Javernick-Will A.-N., Chinowsky P. (2013). The influence of generation on knowledge sharing connections and methods in construction and engineering organizations headquartered in the US. Construct. Manag. Econ..

[79] Ho S.-P., Tserng H.-P., Jan S.H. (2013). Enhancing knowledge sharing management using BIM technology in construction. Sci. World J..

[80] Wang W.-T., Wei Z.-H. (2011). Knowledge sharing in wiki communities: an empirical study. Online Inf. Rev..

[81] Fannoun S., Kerins J. (2019). Towards organisational learning enhancement: assessing software engineering practice. Learn. Organ..

[82] Ochieng E.-G., Ovbagbedia O.-O., Zuofa T., Abdulai R., Matipa W., Ruan X., Oledinma A. (2018). Utilising a systematic knowledge management based system to optimise project management operations in oil and gas organisations. Inf. Techol. & People.

[83] Neeley T.-B., Leonardi P.-M. (2018). Enacting knowledge strategy through social media: passable trust and the paradox of nonwork interactions. Strat. Manag. J..

[84] Nazemi S.-M.-J., Seyed-Hosseini M., Fadaei A. (2011). A game-theoretic approach to knowledge sharing between suppliers. A case study in the Iranian automotive industry (saipa). Australian Journal of Basic and Appl. Sci..

[85] Hutzschenreuter T., Horstkotte J. (2010). Knowledge transfer to partners: a firm level perspective. J. Knowl. Manag..

[86] Roth J. (2003). Enabling knowledge creation: learning from an R&D organization. J. Knowl. Manag..

[87] Landry R., Saïhi M., Amara N., Ouimet M. (2010). Evidence on how academics manage their portfolio of knowledge transfer activities. Res. Pol..

[88] Argote L., Fahrenkopf E. (2016). Knowledge transfer in organizations: the roles of members, tasks, tools, and networks. Organ. Behav. Hum. Decis. Process..

[89] Singh S.-K., Gupta S., Busso D., Kamboj S. (2021). Top management knowledge value, knowledge sharing practices, open innovation and organizational performance. J. Bus. Res..

[90] Levine S.-S., Prietula M.-J. (2012). How knowledge transfer impacts performance: a multilevel model of benefits and liabilities. Organ. Sci..

[91] Barber J., Metcalfe S., Porteous M. (2016).

[92] Da Silva V.L., Kovaleski J.L., Pagani R.N. (2019). Technology transfer in the supply chain oriented to industry 4.0: a literature review. Technol. Anal. Strateg. Manag..

[93] Kretschmer T., Khashabi P. (2020). Digital transformation and organization design: an integrated approach. Calif. Manag. Rev..

[94] Ribeiro-Navarrete S., Botella-Carrubi D., Palacios-Marqués D., Orero-Blat M. (2021). The effect of digitalization on business performance: an applied study of KIBS. J. Bus. Res..

[95] Albukhitan S. (2020). Developing digital transformation strategy for manufacturing. Procedia Comput. Sci..

[96] Fistrić M. (2019). The impact of digitalization on the generation gap–from baby Boomers to generation Z”. Commun. Manag. Review.

[97] Lužar M., Zoran A., Markič M., Bukovec B. (2023). Intergenerational differences and knowledge transfer among slovenian engineers. Organizacija.

[98] Zenk L., Hynek N., Edelmann N., Virkar S., Parycek P., Steiner G. (2022). Exploring motivation to engage in intraorganizational knowledge sharing: a mixed-methods approach. Kybernetes.

[99] Desmidt S., Prinzie A. (2019). Establishing a mission-based culture: analyzing the relation between intra-organizational socialization agents, mission valence, public service motivation, goal clarity and work impact. Int. Publ. Manag. J..

[100] Massaro M., Moro A., Aschauer E., Fink M. (2019). Trust, control and knowledge transfer in small business networks. Review of Manag. Sci..

[101] Gervais M.-J., Marion C., Dagenais C., Chiocchio F., Houlfort N. (2016). Dealing with the complexity of evaluating knowledge transfer strategies: guiding principles for developing valid instruments. Res. Eval..

[102] Correa R.-D., Silva L.-F., Scafuto I.-C. (2023). Mechanisms for capturing and transferring tacit knowledge between projects. Int. J. Knowl. Manag. Stud..

[103] Cairó O., Bork D. (2017). Tacit to explicit knowledge conversion. Cognit. Process..

[104] Nupap S. (2022).

